# Single-Step Purification of Monomeric l-Selectin via Aptamer Affinity Chromatography

**DOI:** 10.3390/s17020226

**Published:** 2017-01-24

**Authors:** Christian Kuehne, Stefanie Wedepohl, Jens Dernedde

**Affiliations:** 1 Institute of Laboratory Medicine, Clinical Chemistry and Pathobiochemistry, Charité-Universitätsmedizin Berlin, 13353 Berlin, Germany; jens.dernedde@charite.de; 2 Institute of Chemistry and Biochemistry, Freie Universität Berlin, 14195 Berlin, Germany; stefanie.wedepohl@fu-berlin.de

**Keywords:** l-selectin, aptamer, DNA, recombinant protein, purification, affinity, SPR

## Abstract

l-selectin is a transmembrane receptor expressed on the surface of white blood cells and responsible for the tethering of leukocytes to vascular endothelial cells. This initial intercellular contact is the first step of the complex leukocyte adhesion cascade that ultimately permits extravasation of leukocytes into the surrounding tissue in case of inflammation. Here we show the binding of a soluble histidine tagged l-selectin to a recently described shortened variant of an l-selectin specific DNA aptamer with surface plasmon resonance. The high specificity of this aptamer in combination with its high binding affinity of ~12 nM, allows for a single-step protein purification from cell culture supernatants. In comparison to the well-established Ni-NTA based technology, aptamer affinity chromatography (AAC) was easier to establish, resulted in a 3.6-fold higher protein yield, and increased protein purity. Moreover, due to target specificity, the DNA aptamer facilitated binding studies directly from cell culture supernatant, a helpful characteristic to quickly monitor successful expression of biological active l-selectin.

## 1. Introduction

Histidine tagged proteins are widely used in recombinant protein production as the purification of His-tagged proteins via Ni-NTA is a versatile and well established method [1,2]. However, to obtain pure and biologically active protein in adequate yields, optimization strategies are commonly necessary. Depending on the quality and quantity of unspecific protein binding from crude mixture to the Ni-NTA matrix, different approaches including competitor based binding and washing buffer compositions (e.g., imidazole, high salt, pH, detergents) are needed, but might reduce the overall yield of the target protein. Protein purification via aptamer affinity chromatography (AAC) is an outstanding alternative, if appropriate [3]. The typically high affinity and specificity of aptamers result in high yields of pure protein due to very low unspecific binding [4,5]. Additionally, genetic engineering to equip the target protein with an affinity tag is dispensable. In contrast, for every new target, a suitable aptamer has to be created. A further problem might be the essential modification of the aptamer, to link the nucleic acid to a solid support. Slight modifications might change the aptamer structure and render the molecule inactive [5,6].

Selectins are a family of membrane bound adhesion receptors that recognize the common tetrasaccharide ligand, sialyl Lewis X. Selectins are involved in the first steps of leukocyte adhesion, which lead to the guidance of immune cells out of the vasculature to sites of inflammation [7]. Neutralization of pathogens is their task in healthy individuals, but in pathological settings such as rheumatoid arthritis or phenomena like reperfusion injury, the migration of leukocytes from the blood stream to the tissues is dysregulated and contributes to damage healthy tissue as well [8]. Here the first step of the adhesion, the tethering of the leukocytes, is a hallmark for the cascade and mainly mediated by the selectins [7,9].

Besides E-selectin that is expressed on activated endothelium and P-selectin that can be found on both activated endothelium and platelets, l-selectin is produced by most leukocytes [10]. The l-selectin specific aptamer LD201 (39 bp) described by Hicke et al. [11] binds in a calcium dependent manner with nanomolar affinity. In 1999 Romig et al. already used a slightly shortened variant (36 bp, LD201mod) of the originally described aptamer for affinity purification of l-selectin IgG fusion protein from CHO cells [12]. An even shorter version of LD201, LD201mΔ1 (28 bp, Table 1), with comparable affinity but significant increase in stability (i.e., increased melting temperature) was recently described [13]. In this study, we used the 5′biotinylated LD201mΔ1 for purification of the monomeric, C-terminal histidine-tagged human l-selectin consisting of the N-terminal Lectin and EGF-like domain (LE-His) from cell culture supernatant. Purification was achieved in a single step. Moreover, calibration-free concentration analysis and affinity determination was possible directly from the crude supernatant by surface plasmon resonance.

## 2. Materials and Methods

### 2.1. Protein Expression

l-selectin LE-His was transiently expressed using the HEK 293-F expression system (Life Technologies, Carlsbad, CA, USA) as described before [14]. Briefly, LE-His inserted in a pcDNA3 vector (Life Technologies, Carlsbad, CA, USA) was used to transfect 500 mL of 293-F cell suspension at a density of 1 × 10^6^ cells per mL. After 72 h of incubation at 37 °C, 8% CO_2_, and shaking at 90 RPM the cell culture supernatant was harvested by centrifugation at 4 °C and 6000× *g* for 20 min and subsequently processed by sterile filtration.

### 2.2. Preparation of the Aptamer Column

550 µg of biotinylated l-selectin aptamer LD201mΔ1 (Metabion, Steinkirchen, Germany) were coupled to 1 mL streptavidin agarose (Thermo Fisher Scientific, Waltham, MA, USA) using PBS with 500 mM NaCl as coupling buffer. Prior to coupling, streptavidin agarose was washed at least five times with coupling buffer before incubation with the aptamer for 2 h at room temperature. Coupling efficiency was monitored by A260 absorption of the supernatant (data not shown).

### 2.3. Purification of l-Selectin LE-His

For comparison three different purification strategies were performed via gravity flow columns at room temperature without the supplementation of DNase inhibitors to the cell culture supernatant to avoid potential aptamer degradation (A: aptamer, B: Ni-NTA, non-optimized, C: Ni-NTA, optimized procedure). Resin capacities were determined using very small column volumes (i.e., 50 µL) and an excess of cell culture supernatant to fully load the resins, keeping unspecific binding at a minimum. The amount of l-selectin bound to the resin was subsequently determined by ELISA. The column volumes were adjusted according to the resin capacities (capacity Ni-NTA: 0.46 mg/mL and aptamer: 0.32 mg/mL; column volume Ni-NTA: 0.695 mL; and aptamer: 1 mL). Columns were equilibrated with an excess of appropriate binding buffer (A, PBS +/+, i.e., with 0.9 mM CaCl_2_, and 0.5 mM MgCl_2_; B, PBS −/−; C, PBS −/− + 20 mM imidazole). Prior to loading the cell culture supernatant to Ni-NTA columns the supernatant was supplemented with 1 mM NiSO_4_ to avoid Ni-ion leaching from the column and for optimized procedure additionally 20 mM imidazole were included. The flow through was collected and the columns were washed with 30 column volumes (CV) washing buffer each (A, PBS +/+; B, PBS −/− + 10 mM imidazole; C, PBS −/− + 500 mM NaCl + 40 mM imidazole). Bound protein was eluted with five CV of elution buffer each (A, PBS −/− + 100 mM EDTA; B and C, PBS −/− + 250 mM imidazole).

### 2.4. Endoglycosidase Digestion

Endoglycosidase digestion with PNGase F was performed as described previously [14]. Briefly, glycosylated l-selectin LE-His was denatured at 95 °C for 5 min in the presence of 1% SDS (*w*/*v*) and 10% 2-mercaptoethanol. After diluting the mixture 1/10, 100 mU PNGase F (Roche Applied Science, Mannheim, Germany) were added and incubated for 16 h at 37 °C.

### 2.5. SDS-PAGE Staining and Western Blotting

The purity of the LE-His protein was analyzed under non-reducing conditions on a 13% polyacrylamide gel and visualized by silver staining. Identity of l-selectin was confirmed by Western blotting with subsequent immunodetection using standard protocols with monoclonal antibody DREG-200 (self-prepared from hybridoma) as primary antibody and a goat-anti-mouse HRP-conjugate (Dako, Glostrup, Denmark) as secondary antibody.

### 2.6. Selectin Quantification via ELISA and SPR

LE-His was quantified by a sandwich ELISA using DREG-200 as capture and biotinylated DREG-55 (self-prepared from hybridoma with subsequent biotinylation) as detection antibody following standard procedures, whereas human serum (Thermo Fisher Scientific, Dreieich, Germany) served as a standard.

Selectin concentration from the cell culture supernatant was additionally determined using calibration free concentration analysis provided by the Biacore X100 with plus package (GE Healthcare, Freiburg, Germany). Here, only the active protein that is able to bind the aptamer is considered. In this set-up, samples are injected at flow rates of 10 µL/min and 100 µL/min under mass transfer limitation. Therefore, biotinylated LD201mΔ1 was coupled to 1900 RU to a streptavidin chip (GE Healthcare, Freiburg, Germany). The reference lane was left untreated. By providing information about the mass (32,114 Da) and the diffusion coefficient (8.5 × 10^−11^ m^2^·s^−1^) of the analyte, the built-in software of the Biacore X100 calculates protein concentrations from the slope differences [15,16,17]. HBS-Ca (20 mM HEPES, pH 7.5, 150 mM NaCl and 1 mM CaCl_2_) was used as running buffer, 100 mM EDTA as regeneration buffer and cell culture medium of untransfected HEK cells was used as a blank.

### 2.7. Affinity Determination via SPR

Binding affinities of l-selectin LE-His to aptamer LD201mΔ1 were measured by kinetic titration series using a Biacore X100 with plus package (GE Healthcare, Freiburg, Germany). A series of five sample dilutions was injected at a flow rate of 30 µL/min over biotinylated LD201mΔ1 coupled to a streptavidin chip (61.4 RU immobilized) (GE Healthcare, Freiburg, Germany) and a mock treated reference lane using running buffer and blanks as mentioned above. Data were analyzed by built-in software of the Biacore X100 [18] providing analyte concentrations as determined by the above mentioned quantification method via SPR.

## 3. Results and Discussion

### 3.1. Protein Purification by Ni-NTA and AAC

For better comparison of Ni-NTA and AAC based purification strategies, column capacities were determined prior to protein clean up. Therefore, small columns (50 µL column volume) were run with an excess of cell culture supernatant (100 mL) to keep unspecific binding to both columns at a minimum (data not shown). Larger columns were run after adjustment of the column volume according to the calculated capacities to match the capacity of each column material.

**Figure 1:**
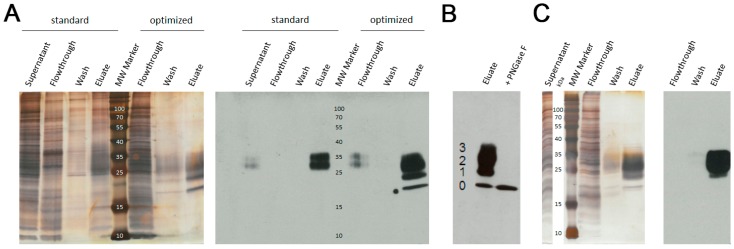
Verification of protein purity and identity after (**A**) Ni-NTA chromatography. Left, silver stained SDS-PAGE and right, respective immunoblot detecting l-selectin LE-His. Standard and optimized purification protocols are compared. The molecular weight (MW) marker is given in kDa; (**B**) Endoglycosidase digest of elute. Numbers indicate the different glycoforms of l-selectin LE-His, 0: no *N*-glycans, 1: one *N*-glycan, 2: two *N*-glycans, 3: three *N*-glycans; (**C**) AAC chromatography. Left, silver stained SDS-PAGE and right, respective immunoblot detecting l-selectin LE-His. The molecular weight (MW) marker is given in kDa.

Purification of the monomeric l-selectin LE-His via Ni-NTA by standard procedures as recommended by the manufacturer (Qiagen, Hilden, Germany) did not produce satisfying results in a one-step purification procedure under standard, un-optimized conditions (Figure 1A). The addition of 20 mM imidazole to the supernatant and column washing with increased salt and imidazole concentration (PBS −/− + 500 mM NaCl + 40 mM imidazole) significantly contributed to shield unspecific binding and resulted in highly pure LE-His protein. In contrast, AAC yielded highly pure l-sel LE-His within one step without the need for further optimization (Figure 1C). Digestion with the endoglycosidase PNGaseF revealed the differently glycosylated isoforms of the l-selectin including the non-glycosylated, fastest migrating protein (Figure 1B) [14].

### 3.2. Aptamer Affinity to l-Selectin

**Figure 2:**
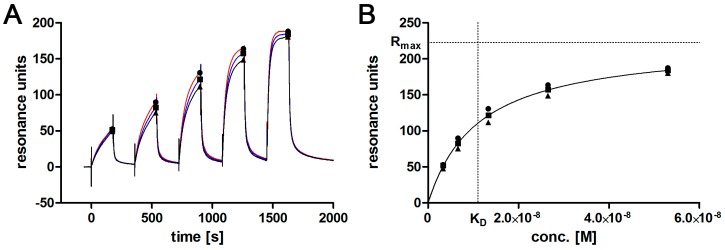
Single cycle kinetics of l-selectin LE-His in cell culture supernatant using SPR. (**A**) triplicate of the kinetic titration is shown. Symbols mark binding levels that will be plotted against the respective concentrations shown in (**B**) binding levels are plotted against corresponding concentrations. Extrapolated R_max_ and respective K_D_ at R_max_/2 are marked by dotted lines.

**Figure 3:**
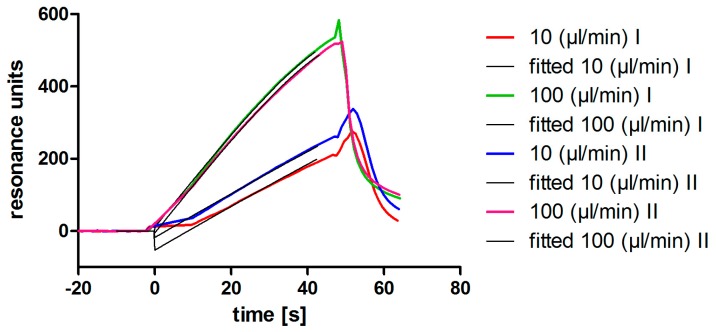
Calibration free concentration analysis using SPR. l-selectin LE-His in cell culture supernatant was analyzed in duplicate. The sensorgrams (colored lines) and the fits (black lines) at the different flowrates are shown.

The originally described aptamer binds to l-selectin with low nanomolar affinity [11]. To determine if the shortened 28 bp variant maintains this high affinity binding, SPR analyses were performed. Ascending concentrations of purified monomeric l-selectin (LE-His) cell culture supernatant were injected in a single run (single cycle kinetics; SCK; Figure 2A) over the biotinylated and immobilized l-selectin aptamer LD201mΔ1, resulting in an affinity of K_D_ = 11.2 ± 0.8 nM (Figure 2B). Related to the original full length aptamer that showed an affinity of 1.8 nM to dimeric l-selectin Fc-chimera in a filter binding assay [11], this is a comparable result. Prior to the affinity measurement, the concentration of l-selectin LE-His in the cell culture supernatant was determined by a calibration free SPR method (calibration-free concentration analysis; CFCA; Figure 3) [15,16,17] yielding a concentration of 200 ± 0.25 nM (SD from duplicate). Interestingly, aptamer degradation by DNase seems not to be a major problem, as recognized by the comparable resonance units obtained during CFCA. Given a total volume of 150 mL and a molecular mass of 32,114 g/mol for the fully glycosylated protein, a total amount of 963 µg was found by CFCA which is in good agreement with 988 µg determined by the ELISA method. Purification via AAC was able to clean up to four-fold the amount of protein compared to the optimized Ni-NTA procedure (Table 2).

## 4. Conclusions

The shortened aptamer variant LD201mΔ1 shows binding comparable to the originally described aptamer [11] that is specifically targeting the leukocyte adhesion receptor, l-selectin, with low nanomolar affinity. The successful application as a blocking agent of leukocyte adhesion [13] and the detachment of the protein from the aptamer by complexation of divalent cations with EDTA strongly suggests a binding mode that involves the Ca^2+^ binding lectin domain. Biotinylation of the DNA aptamer at the 5′ end did not alter target recognition. Its high affinity and selectivity makes the synthetic ligand perfectly suitable to capture l-selectin from crude mixtures like cell culture supernatant. In contrast to standard affinity chromatography approaches for protein purification such as Ni-NTA, AAC does not rely on time consuming optimization procedures and the purified protein can be obtained in a single step in higher yields.

## Figures and Tables

**Table 1:** Sequences of the various shortened variants of the originally described aptamer.

Aptamer	Sequence	Reference
LD201	5′-CAAGGTAACC AGTACAAGGT GCTAAACGTA ATGGCTTCG-3′	Hicke et al. 1996
LD201mod	5′-GCGGTAACC AGTACAAGGT GCTAAACGTA ATGGCGC-3′	Romig et al. 1999
LD201mΔ1	5′-GC · · · · C AGTACAAGGT GCTAAACGTA ATGGC-3′	Riese et al. 2016

**Table 2:** Affinity matrix performance. ^(a)^ amount of l-sel LE-His from cell culture supernatant bound to 1 eq. Ni-NTA or 1 eq. aptamer LD201mΔ1 (ELISA data) ^(b)^ yield calculated by the total amount of l-sel LE-His in 150 mL cell culture supernatant (988 ± 276 µg); ±SD from N = 2 in duplicates.

Affinity Matrix	l-sel LE-His (µg) ^(a)^	Yield (%) ^(b)^
Ni-NTA	167 ± 21	17
LD201mΔ1	608 ± 23	62
